# The Positive Roles for Reactive Oxygen Species in Human Reproduction; Implications for the Therapeutic Application of Antioxidants

**DOI:** 10.3390/antiox15060674

**Published:** 2026-05-27

**Authors:** Emma M. Pyneandee, Hassan W. Bakos, Geoffry N. De Iuliis, Robert J. Aitken

**Affiliations:** 1 Research Centre for Reproductive Science, Life Sciences Building, Discipline of Biological Sciences, School of Science, College of Engineering, Science and Environment, University of Newcastle, Newcastle, NSW 2308, Australia; 2 Memphasys Limited, Sydney, NSW 2140, Australia; 3 Reproductive and Family Health Research Program, Hunter Medical Research Institute, Newcastle, NSW 2305, Australia

**Keywords:** reactive oxygen species (ROS), redox signalling, reproduction, antioxidants

## Abstract

While the pathological impact of reactive oxygen species (ROS) in the aetiology of human infertility has received much attention, this review explores the counterproposal that these highly reactive metabolites play a positive role in mediating reproductive success. The physiological importance of ROS in biological systems can be distilled into three main categories of influence: (1) ROS can oxidize thiols to generate either the corresponding sulfenic acid or disulfide bridges. This oxidizing capacity is critical for several reproductive processes, including the cross linking of sperm chromatin during epididymal maturation, formation of the mitochondrial sheath, and the activation of proteolytic zymogens involved in such processes as ovulation, menstruation, implantation, and parturition. Thiol oxidation is also involved in the suppression of phosphatase activity and the resulting promotion of phosphorylation-dependent signal transduction pathways, which are involved in virtually every aspect of reproduction from sperm capacitation to parturition; (2) The destructive properties of ROS are also biologically significant in the defence against genital tract infections and in mediating such processes as autophagy, apoptosis, and ferroptosis, which are fundamental to the reproductive process; (3) Finally, ROS are involved in controlling the redox status of transition metals (particularly iron and copper) in the active site of many enzymes that are of fundamental importance to reproduction. Given the biological importance of ROS to procreation, we should use antioxidants with care in managing both male and female infertility and avoid the induction of reductive stress.

## 1. Introduction

Oxidative stress (OS) is a well-recognized pathological phenomenon that plays a central role in the aetiology of human infertility. OS can occur as a result of a variety of factors, including: pathological conditions such as varicocele, polycystic ovary syndrome, endometriosis, fibroids, and inflammation [1,2,3], exposure to environmental stressors such as air pollutants, pesticides, and radiofrequency electromagnetic radiation [4,5,6,7], and the impact of a wide variety of lifestyle factors, including diet, obesity, and smoking [8,9,10,11,12]. Fundamentally, OS occurs because of an imbalance between reactive oxygen species (ROS) production and the body’s ability to neutralise those ROS through the action of enzymatic and non-enzymatic antioxidants [13]. This imbalance can lead to cellular damage because their unstable and reactive nature allows ROS to abduct electrons from nearby molecules, including proteins, nucleic acids, and carbohydrates, leading to lipid peroxidation, protein degradation, and the formation of advanced glycation products, respectively. In addition, ROS are instrumental in the activation of cellular defence mechanisms, such as apoptosis/ferroptosis, which can lead to the large-scale depletion of reproductive cells, including the male and female germ lines [12]. Within the microcosm of reproductive health, OS is recognized as having a key role in disrupting such fundamental biological processes such as fertilization, oocyte maturation, ovulation, decidualisation, embryo development, blastocyst implantation, pregnancy, and parturition [13,14,15]. Given all these negative impacts of ROS on the reproductive process, it is reasonable to question why evolution would have encouraged the generation of these potentially toxic metabolites in cells that are so critically important for procreation and transgenerational carriage of the genome. This review addresses the physiological roles of ROS in the reproductive biology of mammals, with particular reference to human fertility and the ability of men and women to generate normal, healthy offspring.

## 2. The Positive Role for ROS in Reproductive Cells

ROS include biologically important signaling molecules that are constantly being generated in cells. Their biochemical significance is built upon three fundamental biochemical principles:

(1) ROS can oxidize cysteine thiols to generate the corresponding sulfenic acid (SOH) or create disulfide bridges, thereby fundamentally altering protein conformation and function. In the following review of the reproductive process, this property of ROS is exemplified in numerous ways, from the cross-linking of chromatin in the sperm head to the activation of zymogens (e.g., metalloproteases) and the suppression of protein phosphatase activity [16,17]. The latter is an extremely powerful consequence of ROS exposure that results in phosphorylation-dependent signal transduction cascades being maintained in an activated state. A reproductively important example is the phosphoinositide 3-kinase (PI3K)/protein kinase B (AKT) pathway, which is essential for cell proliferation, protein synthesis, cell cycle progression, the suppression of apoptosis, the resumption of meiosis, and implantation [18,19]. Similarly, mitogen-activated protein kinase (MAPK) activity is promoted by ROS and drives several processes central to reproductive fitness, including spermatogenesis and oocyte maturation [20,21]. Thiol oxidation may also affect cellular activity via changes in transcription factors, such as Nrf2/Keap1 or NF-κB, that regulate the responsiveness of reproductive cells and tissues to OS and are critical for the survival of the germ line and critical processes, such as ovulation, implantation, and labour [22,23,24]. At higher oxidation states, sulfenic acid residues can generate the corresponding sulfinic acid, or, if the OS is very severe, sulfonic acid; these oxidation products are not readily reversed and are indicative of terminal oxidation and pathology.
(2) At high levels of intensity, ROS can, indeed, be very destructive, attacking a variety of critical biomolecules and inducing physiological cell death [12,25]. This property is an essential element in cellular remodelling processes encountered in reproduction, including luteolysis, menstruation, and implantation, as well as the deletion of defective gametes and embryos. Under these circumstances, ROS is being used as a positive mediator of cellular turnover, driving such key processes as apoptosis, ferroptosis, and autophagy [12,19,26].
(3) A third fundamental property of ROS is that it can interact with metal centres at the core of many key proteins and control redox-switching activities. Typically, such metal centres are occupied by transition metals such as iron and copper, the redox status of which controls the overall function of the protein. For example, the redox regulation of heme-iron centres in key proteins, such as cytochrome P450s, cytochrome C, nitric oxide synthase, catalase, and a variety of peroxidases, is important for diverse reproductive processes such as sperm hyperactivation, progesterone synthesis by the corpus luteum, and the generation of prostaglandins at parturition. Lipoxygenase activity is also dependent on the oxidation of an iron atom at its active site, triggering such vital biological processes as eicosanoid generation and ferroptosis [27]. Similarly, the redox cycling of bound copper supports the functionality of proteins such as superoxide dismutase (SOD) and ceruloplasmin that play critical protective roles at all stages of the reproductive process from gametogenesis to foetal development [28,29].

In the following sections, the positive contributions of ROS to all stages of the reproductive process, from gamete maturation to childbirth, are examined. The review is focused on human reproduction, although the concepts expressed are often supplemented with data obtained from animal models, particularly laboratory rodents. Furthermore, this review focuses on ROS-mediated events and only gives limited consideration to other forms of redox-induced change, including S-nitrosylation, sulfhydration, glutathionylation, CoAlation, and protein carbonylation, which have been considered in detail elsewhere [30]. The results of this analysis clearly illustrate the important biological role of ROS in reproductive biology and highlight why caution should be exercised in determining the type and dose of antioxidants deployed in the therapeutic management of human infertility.

## 3. ROS and Sperm Function

### 3.1. ROS and Chromatin Cross-Linking

**Figure 1:**
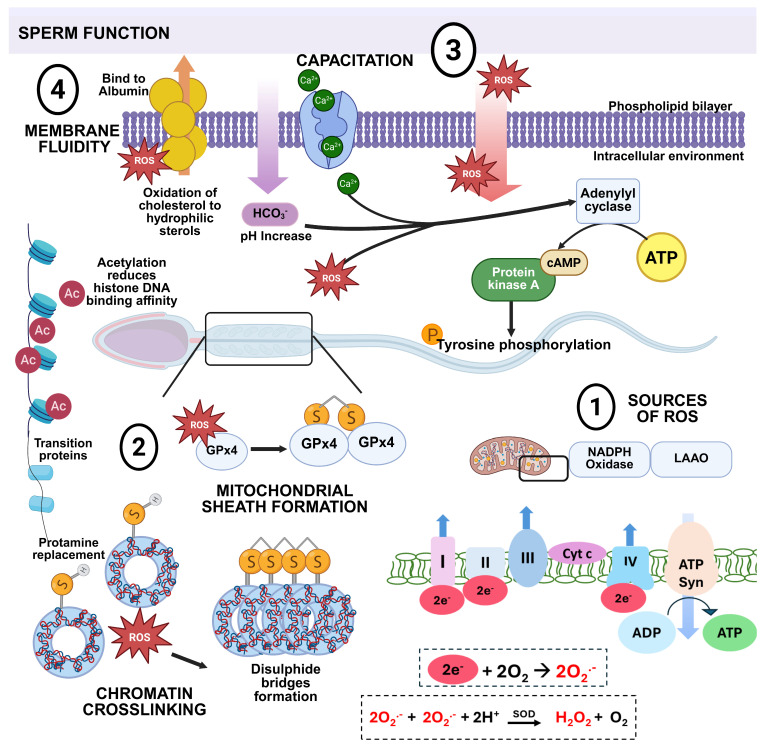
Physiological role of ROS in human sperm function. Step 1. Physiological sources of ROS within spermatozoa include NADPH oxidases, L-Amino acid oxidases (LAAO), and the mitochondria. NADPH oxidases (particularly NOX5) reduce molecular oxygen to superoxide anion (O_2_^•−^) following calcium stimulation, which then dismutates under the influence of superoxide dismutase (SOD) to generate H_2_O_2_. L-Amino acid oxidases (LAAO) generate H_2_O_2_ following exposure to aromatic amino acids such as phenylalanine and tryptophan. The mitochondria leak electrons from the electron transport chain, which are then swept up by molecular oxygen to generate O_2_^•−^. Step 2. During the final stages of spermiogenesis, the cross-linking of glutathione peroxidase molecules mediated by excess H_2_O_2_ leads to stabilization of the mitochondrial sheath. At around the same time, hyperacetylation of histones reduces their affinity for DNA, facilitating their replacement with transition proteins, which are, in turn, replaced by protamines. Protamines are rich in cysteine groups, which become oxidized by ROS during epididymal maturation, leading to the formation of multiple inter- and intra-molecular disulphide bridges that stabilize and compact the chromatin, providing a measure of protection for the DNA. Step 3. Capacitation is characterised by increases in pH, intracellular calcium, HCO_3_^−^, cyclic AMP (cAMP), and protein phosphorylation, all of which are mediated by ROS. Step 4. Hydrogen peroxide oxidises cholesterol, resulting in an increase in hydrophilicity, which enhances oxysterol efflux from the plasma membrane. The resulting increase in membrane fluidity facilitates the anterior movement of egg recognition complexes within the plasma membrane and, following binding to the egg surface, acrosomal exocytosis and sperm-oocyte fusion. (This figure was created in BioRender. Pyneandee, E. (2026) https://BioRender.com/wnbsbft).

Human spermatozoa feature three major physiological sources of ROS, including electron leakage from their mitochondria, the activation of specialized calcium-dependent NADPH oxidases such as NOX5, and the activity of L-amino acid oxidases such as IL4I1 [12] (Figure 1; Step 1). These ROS are then used to drive various aspects of sperm biology, starting with their fundamental architecture. As spermatozoa mature, cysteine groups in an important protective enzyme, glutathione peroxidase 4 (GPX4), become oxidized, leading to the formation of multiple intermolecular disulphide bridges and the formation of a polymer that stabilizes the spiral arrangement of the mitochondria around the axoneme. In this way, an enzyme that started as a powerful antioxidant becomes transformed, via oxidation, to a key structural element in the sperm midpiece, the mitochondrial sheath [31].

Another redox-mediated change in maturing spermatozoa involves chromatin cross-linking in the sperm head. This change compacts the DNA into a near-crystalline state and is thought to be important for safeguarding the integrity of the male genome during its long journey from the male to the female reproductive tract [32,33]. The cross-linking of sperm chromatin is achieved by replacement of nuclear histones with cysteine-rich protamines during spermatogenesis, followed by ROS-mediated formation of inter- and intra- molecular disulfide bridges, mediated by GPX4, during epididymal transit (Figure 1; Step 2) [34,35,36,37]. Although all Eutherian mammals use 6–9 cysteines for disulfide cross-linking, the specific placement and subsequent intra- vs inter-molecular bonding patterns vary. Interestingly, variation in packaging efficiency and DNA integrity appears to correlate with sperm competition levels within a species. Higher competition often selects for more robustly packaged and damage-resistant chromatin to ensure successful fertilisation in a competitive environment. In this context, it is interesting to note that human spermatozoa, which have not evolved to cope with high levels of sperm competition, have notoriously poor chromatin cross-linking and, as a result, are far more susceptible to DNA damage than other Eutherian spermatozoa [36]. Such deficiencies in chromatin packaging correlate with incomplete thiol oxidation during epididymal transit. As a result, spermatozoa from infertile males are often found to possess a higher thiol content (fewer disulfide bonds) compared with normozoospermic, control samples [38,39,40].

### 3.2. ROS and Capacitation

Another crucial pathway regulated by redox-responsive mechanisms is capacitation, the process by which mammalian spermatozoa undergo a final maturation during their ascent of the female reproductive tract and gain the capacity to fertilize the oocyte. Capacitation is characterised by increases in pH, intracellular calcium, HCO_3_, cyclic AMP (cAMP), and protein phosphorylation [41]. ROS are positively involved in many of these processes, as indicated in Figure 1, Step 3. Thus, the increase in tyrosine phosphorylation that characterises the attainment of a capacitated state is a major redox-driven event in human spermatozoa. This enhancement is achieved via the ability of H_2_O_2_ to oxidize catalytic cysteine residues (often protected by glutathionylation or CoAlation) at the active site of tyrosine phosphatases to generate sulfenic acids, which can then react with adjacent cysteines to form disulfide bonds or with nearby amides to form sulfenyl–amide linkages [30,42]. Such oxidation reactions effectively silence phosphatase activity, thereby facilitating the dramatic increase of protein phosphorylation during sperm capacitation.

Additionally, ROS promotes tyrosine phosphorylation by stimulating generation of the second messenger that drives this activity, cAMP, via the ability of O_2_^•−^ to stimulate soluble adenylyl cyclase activity [43,44,45]. While the biochemical mechanisms responsible for redox-regulated cAMP generation in spermatozoa are not fully understood, the adenylyl cyclase within these cells contains cysteine residues that are susceptible to oxidation, forming disulfide bonds or sulfenic acids. These modifications may induce a structural shift in the catalytic domain, increasing the enzyme’s affinity for its substrate, ATP, or its essential cofactors, Mg^2+^ or Mn^2+^. In addition, ROS facilitates capacitation by enhancing the rate of cholesterol efflux from the plasma membrane, thereby contributing to an increase in membrane fluidity [46]. This phenomenon reflects the ability of ROS to directly induce the formation of oxysterols, which are more hydrophilic than the parent sterols and, as a result, move closer to the sperm surface where they are removed by binding proteins such as albumin (Figure 1; Step 4). The disruption of this redox process using hydrophobic antioxidants, such as vitamins E or A, suppresses the tyrosine phosphorylation events associated with sperm capacitation and also interferes with sperm–zona interaction [47].

Hyperactivated motility is another feature of capacitation that is redox regulated. This type of movement is characterised by a change in the flagellar waveform to deliver a high-amplitude, high-intensity, asymmetrical beat pattern. This vitally important change is dependent on associated changes in tyrosine phosphorylation and is stimulated by ROS via the MAPK/ERK (Extracellular Signal-Regulated Kinase) pathway [48,49,50]. Another ROS-regulated factor involved in the control of hyperactivation is the Epidermal Growth Factor Receptor (EGFR), a transmembrane protein found in many cell types that mediates differentiation, cell proliferation, and migration. EGFR activity has been shown to be directly enhanced by ROS through the inhibition of the receptor’s internalization and, indirectly, via the suppression of protein phosphatase activity [51,52,53]. In spermatozoa, ROS-dependent EGFR activation leads to actin polymerization, which, in turn, leads to hyperactivated motility by mechanically facilitating flagellar bending and acting as a regulatory scaffold for key signalling molecules such as Catsper [54,55,56]. In keeping with this model, studies have shown that light-induced ROS production successfully activates EGFR, leading to hyperactivated motility, contrasting with the suppression of this process by the presence of SOD [54].

Taken together, these data clearly indicate that the physiological generation of ROS, particularly H_2_O_2_, plays a critical role in the orchestration of human sperm form and function, in the lead up to fertilization.

### 3.3. ROS and Fertilization

Following successful hyperactivation and capacitation, further redox-regulated changes lead to egg recognition, the acrosome reaction, and, finally, sperm–oocyte fusion [41,45,55,56]. The ROS-mediated increase in membrane fluidity during capacitation enables egg-recognition complexes to move anteriorly within the plasma membrane to a location where sperm–zona recognition can be achieved [57]. This cell–cell interaction then activates signal transduction processes in the spermatozoa, leading to the induction of the acrosomal exocytosis and, ultimately, fusion with the vitelline membrane of the egg.

The mechanistic underpinnings of these membrane fusion events appear to involve the ability of ROS to induce lipid peroxidation via the lipoxygenase pathway, followed by activation of phospholipase A2. The latter then enzymatically removes the oxidized fatty acid from position 2 (*sn*2) of the phospholipid, generating a lysophospholipid [58,59]. Possessing just one fatty acid tail, these molecules possess detergent-like properties that promote membrane instability, thereby supporting fusion of the plasma and outer acrosomal membranes during acrosomal exocytosis. ROS might also be involved in the IP3-mediated activation of actin severing proteins that promote the acrosome reaction by further destabilizing the plasma membrane [54,55]. The increase in membrane instability might also explain the observed positive role of ROS in the generation of a fusogenic equatorial segment in the spermatozoa, primed for interaction with the oocyte’s plasma membrane [60].

### 3.4. ROS and Sperm Vitality

Finally, ROS are also involved in the mediation of both sperm survival and senescence. In the context of sperm vitality, ROS has been suggested to act directly on AMP kinase (AMPK), activating this enzyme via the oxidation of key cysteines (299 and 304) on the α-subunit and upregulating both the cells’ antioxidant defences, as well as their capacity for ATP generation [61,62]. On the other hand, ROS are also central to the induction of apoptosis in human spermatozoa, facilitating the apoptotic removal of aged, damaged spermatozoa and ensuring that they do not participate in the fertilization process [63]. In light of these observations, it has been suggested that sperm capacitation and senescence represent a physiological continuum mediated by ROS [64]. During the early stages of sperm capacitation, ROS is a positive mediator of biological change, promoting signal-transduction pathways, elevating intracellular calcium, and increasing membrane fluidity under the protective control of antioxidants, notably peroxiredoxin-6 (PRDX6) [65]. However, if capacitated spermatozoa do not find an oocyte, these same changes will ultimately generate senescence and seal their apoptotic fate [64].

## 4. ROS and Oocyte Function

### 4.1. Oocyte Recruitment and Maturation

**Figure 2:**
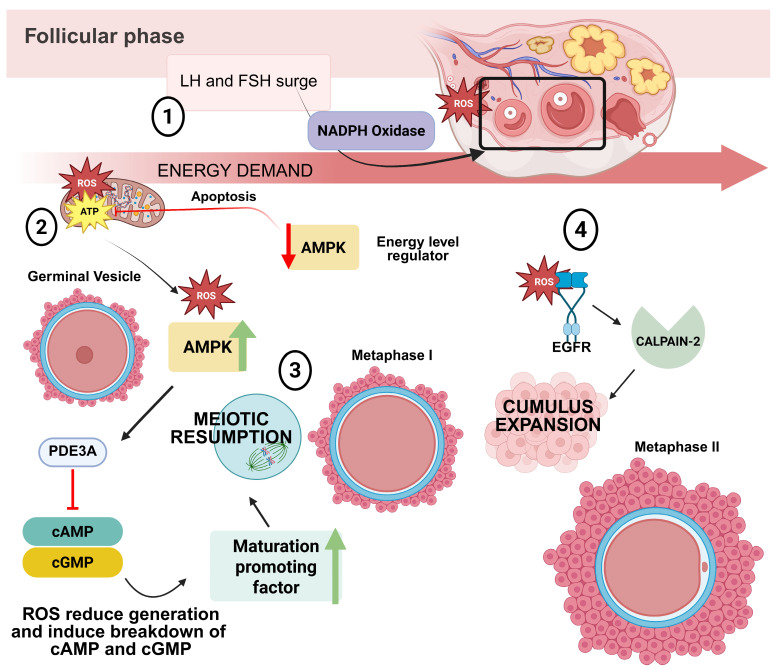
Redox-signalling pathways involved in oocyte maturation during follicular phase. Step 1. The gonadotrophin surge that characterises the end of follicular phase triggers NOX enzymes in the granulosa and thecal cells to produce ROS. Step 2. The increase in energy demand of the developing follicle results in an increase in mitochondrial activity with an increase in electron leakage and, as a result, the further generation of ROS. Step 3. At this point, ROS acts as part of a negative feedback loop by activating AMPK, a marker of adequate energy production. Physiological levels of ROS maintain the level of activated AMPK and promote mitochondrial activity. Activated AMPKstimulates PDE3A (phosphodiesterase 3A) activity, facilitating the reduced generation and accelerated breakdown of cAMP and cGMP. This leads to the activation of maturation-promoting factor, triggering the resumption of meiosis. Conversely, a depleting pool of available ROS decreases AMPK signalling, triggering the apoptotic pathway and cell death. Step 4. ROS mediates the activation of EGFR and thereby modulates the calpain-2 pathway, partly responsible for cumulus mucification and expansion. (This figure was created in BioRender. Pyneandee, E. (2026) https://BioRender.com/orf6chz).

Oocyte recruitment, development, and maturation involve a series of complex molecular signalling processes within the follicular microenvironment, many of which are mediated by ROS [66]. Oocytes spend much of their lifespan reposing within primordial follicles and arrested at prophase I. Throughout this prolonged period of time, they are protected from OS because the oocyte mitochondria lack Complex 1, the major site of electron leakage and ROS generation in a majority of cell types [67]. Following follicle selection and growth, a pre-ovulatory surge of gonadotrophins (LH and FSH) triggers ROS production by granulosa cells in the follicle, precipitating germinal vesicle breakdown (GVB) and meiotic resumption (Figure 2; Step 1). As germinal vesicle breakdown occurs, there is a massive redistribution of mitochondria within the oocyte so that they come to aggregate around the meiotic spindle, which is an ideal position to provide the localized ATP required for spindle assembly, chromosome alignment, and polar body extrusion. In association with germinal vesicle breakdown, oocyte mitochondria acquire Complex 1, along with Complex IV and citrate synthase, at levels comparable to skeletal muscle. As the oocyte maturation process progresses, mitochondrial membrane potential increases, ATP production is accelerated, oxygen consumption is enhanced, and the baseline generation of ROS is elevated [68]. Mitochondria, therefore, appear to be a major physiological source of ROS in maturing oocytes.

Within the whole cumulus-oocyte complex (COC), however, the mitochondrial contribution to redox-regulated oocyte maturation is reinforced by NADPH oxidase activity, particularly NOX4. Thus, FSH-induced maturation of COCs involves translocation of two cytosolic components of NOX4, p47phox, and p67phox to the plasma membrane of cumulus cells, where they activate the oxidase, generating ROS [69]. Simultaneously, ATP production by the cumulus cell mitochondria rises to meet the energetic demands of oocyte maturation, with ROS produced as a by-product (Figure 2; Step 2) [70]. Under physiological circumstances, the integrated contributions of mitochondria and NOX4 generate sufficient ROS within the COC to activate signalling pathways such as AMPK and EGFR, which are involved in energy sensing, cumulus expansion, and ovulation. Both ROS-induced activation of AMPK and calcium-signalling pathways have been implicated in meiotic resumption from diplotene arrest (Figure 2; Step 3) [71,72].

LH also stimulates ROS production in the ovary, as suggested by ascorbic acid depletion studies [73], and this transient OS is functionally important, inducing phosphorylation and activation of the EGF receptor, as well as its downstream effector, p42/44 MAPK (Erk2 and Erk1). These ROS-mediated changes are causally involved in normal cumulus expansion and mucification. Thus, H_2_O_2_ exposure can fully mimic the effect of LH, bringing about an extensive mucification/expansion of the follicle-enclosed cumulus–oocyte complexes. In addition, oocyte maturation can be effectively inhibited in vitro in both mice and rats by membrane-permeant antioxidants such as BHA (butylated hydroxyanisole) [74,75].

A core component of oocyte maturation occurs when ROS-activated AMPK participates in the activation of phosphodiesterase 3A (PDE3A) activity, thereby facilitating reduced generation and accelerated breakdown of cAMP and cGMP. The decline in cAMP, in particular, leads to dephosphorylation of maturation-promoting factor (MPF) and resumption of meiosis. However, if the metabolic activity of oocytes fails to meet mitochondrial thresholds, as reflected in poor AMPK activation, then ROS-mediated apoptotic pathways are initiated by cytochrome C release, inducing large-scale cell death and a state of follicular atresia (Figure 2. Step 3) [75]. In addition to ROS-mediated activation of AMPK, EGFR activation by H_2_O_2_ stimulates the proteolytic calpain-2 pathway, leading to loosening of granulosa cell adhesions, thereby allowing these cells to expand and reorganise as the follicle grows [76] (Figure 2; Step 4).

Ovarian follicles are, therefore, much like spermatozoa in that they rely significantly on redox regulation. ROS-signalling pathways are crucial for the stimulation of follicle growth and the resumption of meiosis. While ROS are potentially toxic, the short half life and limited diffusion capacity of these molecules helps confer spatial precision to these signalling events within the ovarian follicle. However, if ROS generation does overwhelm the follicle’s defensive capacity, then these same metabolites can trigger pathways leading to apoptosis and cell death. Thus, the ultimate fate of each follicle, and the oocyte it encases, critically depends upon its capacity to manage ROS, divining whether it will successfully achieve ovulation or succumb to atresia [77,78,79,80].

### 4.2. ROS and Ovulation

**Figure 3:**
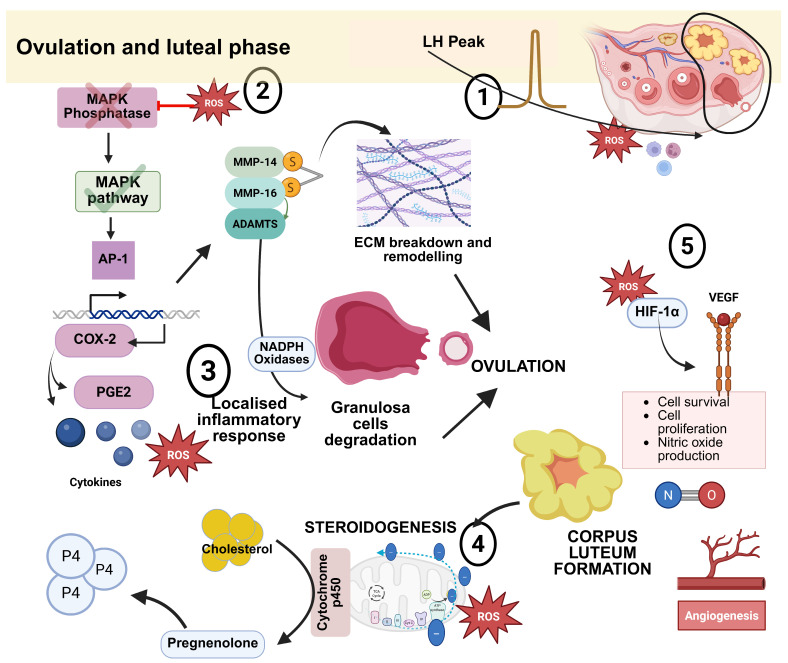
ROS as mediators of ovulation and the luteal phase. Step 1. ROS production by mitochondria and NOX enzymes is triggered by the pre-ovulatory LH peak. The concomitant generation of vasoactive mediators (prostaglandins, histamine) and chemokines also triggers the recruitment of inflammatory cells, which furthers the induction of an oxidative state. Step 2. ROS inhibits MAPK phosphatase activity, which promotes the MAPK pathway, leading to the generation of transcription factors, such as AP-1, that drive the further expression of MMPs. Step 3. ROS activates COX-2, leading to production of PGE2. The latter induces cumulus expansion and furthers upregulation of MMPs that act on granulosa cells and facilitate degradation of the extracellular matrix in the follicle wall, facilitating its rupture. Step 4. Post-ovulation, the evacuated follicle transforms into a corpus luteum (CL) that generates progesterone (P4). ROS facilitates this branch of steroidogenesis by activating a cholesterol side-chain cleavage enzyme (Cytochrome P450), which requires electrons donated from the mitochondrial electron transport chain to facilitate the generation of pregnenolone, the P4 precursor. Step 5. The development of a healthy corpus luteum requires cell proliferation, as well as angiogenesis. During this process, ROS stabilises HIF1a, driving expression of VEGF6, which, in turn, leads to the production of nitric oxide (NO), which enhances blood flow and, hence, the release of P4 into the circulation. (This figure was created in BioRender. Pyneandee, E. (2026) https://BioRender.com/wnbp4on).

Ovulation is similarly modulated by redox activity. As indicated above, the LH surge that precedes ovulation is associated with a sharp rise in inflammatory precursors in the ovary and an increase in ROS generation (Figure 3; Step 1) [81]. A causal relationship between acute inflammation, ROS production, and ovulation has been suggested by several lines of evidence [82,83,84]. Critical in this regard is the ability of ROS generated by the granulosa cells to promote the activation of proteases (matrix metalloproteinases, MMPs-14 and 16, and plasminogen activators) that actively digest the collagen and connective tissues of the follicular wall (the theca externa), creating a weakened area called the stigma, which ultimately becomes the point of follicular rupture (Figure 3; Step 2). MMP activation involves a cysteine switch whereby ROS oxidise a thiol group in the pro-domain of the inactive zymogen, displacing a zinc ion and inducing enzyme activation. ROS also promotes MMP by inhibiting key phosphatases, allowing activation of the MAPK pathway and facilitating the stimulation of transcription factors such as activator protein-1 (AP-1), which bind to the promoter region of MMP genes [16,17,84,85]. Such redox-regulated protease activation, in conjunction with ROS-induced apoptosis of granulosa cells, promotes detachment of the cumulus cells from the follicular wall, further contributing to follicular wall breakdown and ovulation of the mature oocyte [84]. LH also triggers the activation of EGFs (Epidermal Growth Factor-like factors) in granulosa cells via ROS-mediated activation of MMPs [86]. The EGFs then mediate the action of LH in the ovarian follicle [75].

The physiological sources of ROS in mural granulosa cells primarily involve NOX4, possibly under the influence of pigment–epithelium-derived factor (PEDF) [87], as well as electron leakage from the mitochondria [88]. In addition, ROS may also be generated via a similar leakage of electrons from the cyclooxygenase-2 pathway during the biosynthesis of PGE2 [89] (Figure 3; Step 3). In concert, these various sources of ROS combine together to generate the redox drive for follicular rupture and ovulation [90]. Animal studies using *Drosophila* support the involvement of ROS in ovulation by demonstrating that O_2_^•−^ generating NOX enzymes work in conjunction with SOD3 to generate H_2_O_2,_ which then acts as a secondary messenger triggering the apoptotic pathways that lead to follicular rupture [91]. Moreover, in vivo studies in rodents, as well as ex vivo studies in the rabbit, also corroborate the essential role for ROS in follicular rupture and ovulation [75,82,87,91].

The generation of ovulatory estrogen is another crucial function of the Graafian follicle. According to the two-cell/two-gonadotrophin theory, LH stimulates follicular thecal cells to generate androgens, which are then converted into estrogen by the granulosa cells under the influence of FSH. This is a redox-regulated process instigated by cytochrome P450 side-chain cleavage activity within the mitochondria to actively convert cholesterol to pregnenolone, and, ultimately, androgens, within the thecal layer. These events depend on the movement of electrons from the mitochondrial electron transport chain (ETC) to the P450 enzyme, mediated by adrenodoxin and the NADPH-dependent FAD flavoprotein, adrenodoxin reductase (Figure 3; Step 4). This reliance on electron transfer elevates the risk of ROS production [92], which is, in turn, quickly controlled by peroxidase, catalase, and other non-enzymatic antioxidants to ensure that an appropriate redox balance is maintained [68,69]. As antral follicles expand, the thecal layer also becomes heavily vascularized to optimize the uptake of substrates (Low- and High-Density Lipoproteins), as well as the subsequent export of estrogen into the general circulation. In this situation, mitochondrial ROS facilitates stabilization of Hypoxia-Inducible Factor 1-alpha (HIF-1α) that then upregulates Vascular Endothelial Growth Factor (VEGF) in thecal cells, promoting the vascular expansion essential for dominant follicle survival.

## 5. ROS, Corpus Luteum Function, and Menstruation

Following ovulation, the corpus luteum (CL) is formed from the residual follicular remnants and is tasked with producing progesterone to delay the shedding of the endometrium, thereby allowing implantation to occur. Nitric oxide (NO) promotes blood flow and angiogenesis within the newly formed CL. In a similar fashion to neoangiogenesis within the thecal layer during follicle development, low levels of mitochondrial ROS generation also stabilize HIF-1α, leading to the enhanced generation of VEGF, which then drives the massive influx of blood vessels into the CL (Figure 3; Step 5) [93]. At the same time, ROS is also central to luteal steroidogenesis through its support of cytochrome P450 activity.

Whilst ROS and RNS (reactive nitrogen species) are crucial in establishing the corpus luteum, there is a simultaneous shift towards the upregulation of antioxidant protection. Thus, the CL, literally the “yellow body”, acquires its colour from the presence of a powerful antioxidant, β-carotene, designed to limit the amount of oxidative damage incurred by the CL during the luteal phase. The intracellular ROS responsible for creating such stress are just byproducts of the luteal cell’s intense steroidogenic activity and emanate largely from the mitochondrial cholesterol side-chain cleavage activity (particularly Cytochrome P450 Family 11 Subfamily A Member 1; CYP11A1). Nevertheless, the presence of abundant β-carotene, alongside classical antioxidant enzymes such as SOD, catalase, and GPX, ensures that ROS is effectively scavenged during the lifespan of the CL in order to prolong the generation of progesterone.

However, in the absence of a conceptus, SOD expression ultimately decreases and ROS accumulates, leading to the initiation of luteolysis [94,95]. During this process, ROS uncouples the LH receptor from adenylyl cyclase and inhibits steroidogenesis by interrupting transmitochondrial cholesterol transport. ROS (particularly lipid peroxides) also activates the cyclooxygenase (COX-2) responsible for regulating the production of prostaglandin F2α (PGF2α) and triggers the induction and expression of a transcription factor, nuclear factor-kappa B (NF-κB), that binds to the promoter region of the *PTGS2* gene encoding COX-2, dramatically enhancing PGF2α synthesis [95,96]. The latter then acts on both luteal cells and inflammatory cells, such as macrophages and neutrophils, in the immediate vicinity, prompting them to upregulate ROS production in a self-perpetuating cycle to complete the destruction of the CL in preparation for the initiation of another menstrual cycle.

In parallel with luteolysis, ROS is also a mediator of the endometrial regression that characterises menstruation. In this context, the fall in progesterone levels resulting from a lack of human chorionic gonadotrophin (hCG) signalling from the fetus creates a pro-inflammatory state that activates NF-κB and triggers the expression of several genes with a key role in menstruation, including COX-2, MMPs, and cytokines [97,98]. The latter then triggers the infiltration of leukocytes and the generation of more ROS. Oxidative stress, exacerbated by local hypoxia caused by PGF2α-induced vasoconstriction, triggers widespread apoptosis in the upper layer of the endometrium (stratum functionalis), allowing endometrial shedding while minimizing damage to the underlying tissues and preventing development of a chronic inflammatory condition that might damage future fertility [97,98,99].

## 6. ROS Involvement in the Establishment of Pregnancy

### 6.1. ROS and Early Embryonic Development

Following ovulation, ROS are centrally involved in the rapid senescence of the unfertilized oocyte, thereby ensuring that an ageing egg is rapidly eliminated and cannot contribute a potentially damaged genome to the next generation [100]. If fertilization does occur, physiological concentrations of ROS are involved in pronuclear formation, initiation of cleavage, and subsequent cell proliferation [101,102]. However, it is important to note that the needs of the embryo change as embryo development progresses, and, therefore, reasonable to infer that ROS production would also vary. During the cleavage stages of embryonic development, energy production relies on the oxidation of pyruvate via carboxylic acid metabolism, with lactate being produced as a by-product [101,102]. At this stage of embryogenesis, ROS production is relatively low, and the embryo is extremely vulnerable to OS. This offers a mechanistic explanation for the limitations experienced in early embryo culture systems, including the two-cell block in mice and the four-cell block in human embryos [103,104]. Such blocks occur at the moment of zygotic genome activation due to ROS accumulation in the high-oxygen-tension environments employed for embryo culture, impacting effective activation of the embryonic genome and thus hindering cellular division [105,106].

**Figure 4:**
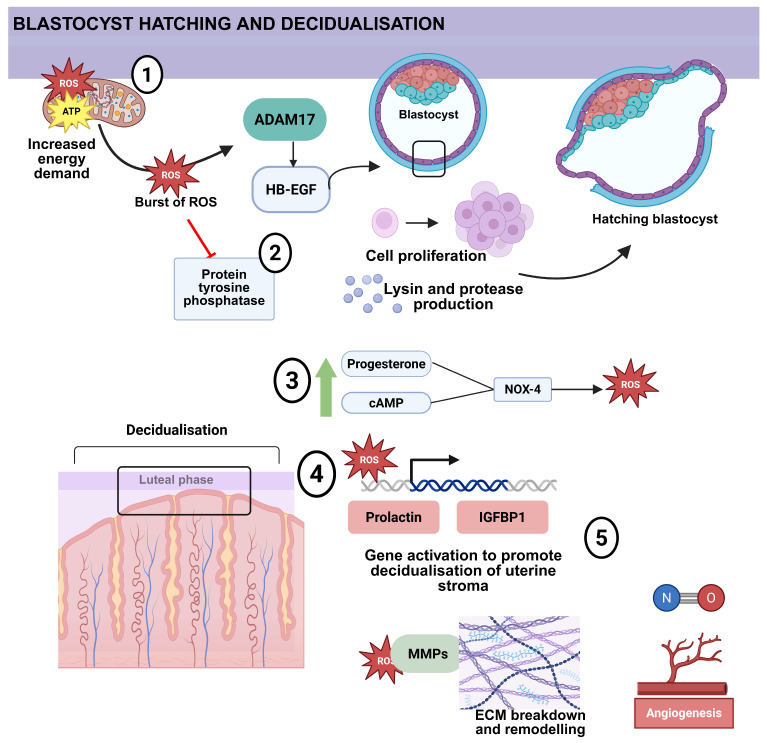
Impact of ROS signalling on blastocyst hatching and decidualisation of uterine stroma. Step 1. The implanting blastocyst exhibits increased O_2_^•−^ production as a result of increased metabolic activity, and, potentially, NADPH- and xanthine oxidase activities. Step 2. ROS inhibit the activity of protein tyrosine phosphatase and promote that of ADAM17, allowing for the release of HB-EGF. HB-EGF acts on the trophectoderm of the blastocyst, promoting cell proliferation and secretion of proteases. The combined effect of blastocyst expansion and lytic secretions contributes to the weakening of the zona pellucida and, subsequently, the hatching of the blastocyst. Step 3. In the uterine stroma, increases in progesterone and cAMP drive decidualisation by firstly activating NOX4 and triggering ROS production. Step 4. The resulting signalling cascade includes gene activation necessary to promote decidualisation of the uterine lining. Step 5. This stage includes the activation of MMPs that work towards degrading the endometrial ECM to assist trophoblast invasion. The syncytiotrophoblast cells also express nitric oxide synthase to increase the production of NO and promote angiogenesis. (This figure was created in BioRender. Pyneandee, E. (2026) https://BioRender.com/pjk2859).

When the embryo develops into a blastocyst, the mitochondria increase their contribution to overall metabolism, and ROS generation is accelerated due to electron leakage from the ETC (Figure 4; Step 1). The ROS generated at this time influence patterns of gene expression, generating proteins that support embryo growth and differentiation [107,108]. Central to this developmental response to OS is the ability of ROS to activate signal transduction pathways such as MAPK, which is centrally involved in the process of lineage determination [109,110,111]. Through their ability to cross-link cysteines on the redox-sensing protein KEAP1, ROS are also able to stabilise the transcription factor NRF2, which regulates genes involved in maintaining redox homeostasis [112]. Other transcription factors important for development, including hypoxia-inducible factors and proteins in the FoxO subfamily, are also responsive to ROS and, again, play a key role in ensuring that the developing embryo does not suffer from OS [113]. The physiological generation of ROS in the early embryo largely involves NOX, complexes I-III of the mitochondrial ETC, and xanthine oxidase, while superoxide dismutase, catalase, glutathione peroxidase, and peroxiredoxins are the major defences against oxidative damage [113,114,115,116].

In addition to their role in embryonic cell division and differentiation, ROS are also a powerful mediators of cell death, ensuring that damaged cells do not contribute to the developing embryo and that defective embryos do not contribute to future generations. The use of ROS in the apoptotic deletion of defective cells is, therefore, a quality-control measure ensuring that the maternal investment in pregnancy will ultimately be productive.

### 6.2. ROS and Implantation

Implantation involves synchronised interactions between the trophoblast, derived from the trophectoderm of the blastocyst, and the epithelial lining of the uterus. In humans, the process of implantation involves three basic phases: apposition of the activated and hatched blastocyst to the endometrial lining, adhesion, and finally invasion of the decidualised endometrial lining. The decidual cells regulate invasion of the blastocyst through immune responses and paracrine signalling, as well as other molecular signalling pathways. Mounting evidence suggests that physiological concentrations of ROS contribute to the cellular and molecular events underlying the implantation process [117,118].

Once formed, the blastocyst must hatch out of the zona pellucida before implantation can occur. The vigorous pulsing activity of the blastocyst at the time of hatching necessitates a sudden increase in mitochondrial ATP production, which, in turn, leads to a spike in local O_2_^•−^ production as a consequence of electron leakage from the mitochondria, with possible additional input from NADPH and xanthine oxidase activities [119,120] (Figure 4; Step 1). Thomas et al. demonstrated the pivotal role of ROS, specifically O_2_^•−^, plays in blastocyst hatching in the mouse [119]. Their studies on murine embryos involved comparison of blastocysts in pre-, peri-, and post-hatching stages. While blastocysts in both pre- and post-hatching stages recorded low levels of O_2_^•−^ and high SOD activity, blastocysts in the peri-hatching stage showed the opposite. The addition of SOD to peri-hatching blastocysts in vitro decreased hatching, while direct exposure of such embryos to O_2_^•−^ enhanced this process [119]. ROS generated by this oxidative burst activated a family of enzymes called ADAMs (A Disintegrin and Metalloproteinases), particularly ADAM17, which cleaved pro-HB-EGF (Heparin-binding epidermal growth factor-like growth factor) to release the biologically active molecule, HB-EGF (Figure 4; Step 2). The latter then promoted the rapid proliferation of trophectoderm cells and the accumulation of fluid within the blastocoel. The resultant increase in internal hydrostatic pressure physically stretches the ZP from the inside, forcing this structure to fracture. HB-EGF signalling also triggers the blastocyst to produce and secrete lysins or proteases that chemically digest and weaken the ZP, making it easier for the embryo to escape. ROS also silence protein tyrosine phosphatases, which would otherwise suppress HB-EGF signalling by dephosphorylating the cognate receptor (EGFR) [121,122,123,124,125].

A hatched blastocyst must then engage in a series of interactive events with the endometrium to allow for successful implantation, such as apposition, adhesion/attachment, and invasion. Hormone synthesis, inflammatory-like events, uterine secretions, and gene expression all have a part to play in orchestrating these critical changes, allowing for interactions between the endometrial lining and the blastocyst [125]. During apposition, the uterine wall secretes lysophosphatidic acid (LPA), the production of which involves the oxidation of low-density lipoprotein (LDL) by ROS [126]. LPA, in turn, triggers a downstream signalling pathway involving G protein-coupled receptors and phospholipase C, leading to the release of Ca^2+^ and activation of protein kinase C (PKC). This process culminates in the shedding of embryonic HB-EGF into the uterine environment, signalling the blastocyst’s presence [127]. LPA also drives COX-2-derived prostaglandin E_2_ (PGE_2_) production in the luminal epithelium and the stroma at the site of adhesion. In addition, LPA increases nitric oxide synthase activity, leading to the production of NO at the implantation site [127,128]. The combination of PGE_2_ and NO plays a key role in angiogenesis, leading to the evolution of a highly vascularised nidus capable of supporting implantation.

Decidualisation of the endometrial cells is another important facet of implantation, characterized by the morphological remodelling and differentiation of maternal stromal cells during the secretory phase of the menstrual cycle. Decidualisation is mediated by oestrogen, progesterone, transcription factors, cytokines, and other complex signal-transduction pathways. This process sees the elongated, fibroblast-like cells of the uterine stroma transforming into rounded, epithelioid-like cells. Interestingly, decidualisation not only involves a morphological transformation of existing stromal cells but is also associated with the influx of inflammatory cells into the uterus prior to blastocyst adhesion [128,129]. The initiation of decidualization is a biphasic process involving ROS [130]. While progesterone and cAMP are the primary drivers, they use ROS to activate certain genes. Thus, decidualization triggers the activation of a free radical-generating NADPH oxidase (NOX4), producing a burst of O_2_^•−^ and H_2_O_2_ inside the cell, which is needed to activate the transcription factor C/EBPβ (CCAAT/enhancer-binding protein beta) (Figure 4; Step 3). This factor then binds to the promoters of key decidual markers like prolactin and insulin-like growth factor-binding protein-1 (Figure 4; Step 4) [130,131]. Significantly, antioxidants or NOX inhibitors can block the generation of ROS during this initiating phase of decidualization, reducing the expression of decidual markers.

In addition to this positive role for ROS in initiating the decidualisation process, the formation of these cells is associated with the upregulation of antioxidant enzymes, like SOD2, catalase, glutathione peroxidase, and glucocorticoid-inducible kinase-1 [132,133]. This sudden elaboration of defensive enzymes designed to protect against oxidative damage does not impair the ability of ROS to drive positive physiological changes during implantation but rather helps prepare the uterus for the looming oxidative burden associated with pregnancy. 

The final step of implantation is the invasion of the endometrial lining by the blastocyst (Figure 4; Step 5). To accomplish this step, the trophoblast layer differentiates into different subtypes: villous cytotrophoblasts (vCTBs) and syncytiotrophoblasts (STs). The invasive behaviour and functional characteristics of cytotrophoblasts have often been compared to those observed in malignant cells, especially when considering the expression of Tubulointerstitial nephritis antigen-like 1 (TINAGL1 or lipocalin7), a structural matrix protein that interacts with integrins on the trophoblast surface and endometrial epithelium, enabling adhesion [134]. This adhesion event then triggers intracellular signalling cascades, including the FAK (Focal Adhesion Kinase) and MAPK/ERK pathways, which are essential for trophoblast outgrowth and promoted by the local generation of ROS, through the latter’s ability to inhibit tyrosine phosphatases [135]. Cytotrophoblasts, at the time of invasion, also exhibit a downregulation of E-cadherin, a negative regulator of trophoblast invasion. By analogy with cancer cell invasion, the generation of ROS is thought to trigger this change in E-cadherin expression through the upregulation of transcription factors such as Snail (SNAI1) and Slug (SNAI2), which are transcriptional repressors of the *CDH1* gene (which encodes E-cadherin), as well as stimulation of the MAPK/ERK kinase pathway [136,137,138]. ROS can also stabilize Hypoxia-Inducible Factor 1-alpha (HIF-1α), which further promotes the expression of E-cadherin repressors to facilitate trophoblast invasion [139,140]. During the invasive stage of embryo implantation, the syncytiotrophoblast expresses endothelial nitric oxide synthase to increase the production of NO, a vasodilator, which is of critical importance in establishing the placentation process [140]. Trophoblast invasion is also facilitated by a range of MMPs (MMP-1, MMP-2, MMP-3 MMP-9, MMP-11, and MMP-14) that have been identified in human placentae [141,142]. ROS is critical for the activation and production of MMPs via pathways that involve the upregulation of MAPK and PI3K/Akt [143,144,145]. These enzymes are central to the implantation process, promoting degradation of the ECM and allowing the trophoblast to break through the uterine lining.

## 7. ROS and Parturition

The fertility journey culminates in parturition, a complex process featuring hormonal fluctuation, inflammatory reactions, and rapid tissue remodelling. ROS are physiologically involved in several aspects of parturition, including the myometrial contractions that initiate labour and the ripening of the cervix, largely through activation of the MAPK pathway. P38 MAPK proteins, modulated by ROS, initiate the release of pro-inflammatory cytokines and prostaglandins from the uterine lining [146]. Within the same tissues, oxytocin activates NF-κB-mediated inflammatory-signalling pathways, leading to stimulation of ROS generation via NADPH oxidases (primarily NOX 1 and NOX4), the sensitisation of oxytocin receptors, and the onset of myometrial contractions, leading to labour [147,148]. Further evidence of the crucial role of ROS during parturition can be seen in the ability of non-enzymatic antioxidants to reduce the risk of preterm birth [148]. In this context, N-acetylcysteine was found to decrease COX-2 and prevent the activation of NF-κB by scavenging local ROS generation and inhibiting some of the key downstream-signalling pathways involved in the onset of labour [149].

## 8. Physiological ROS, Reductive Stress, and Antioxidant Therapy

Given that ROS play such an important role in the biology of reproduction, it would be reasonable to ask whether the indiscriminate administration of antioxidants, albeit with the best of therapeutic intentions, might impede rather than promote this process. The oversupplementation of healthy individuals who are not suffering from OS with antioxidants runs the risk of impairing the myriad cellular-signalling pathways that rely on physiological levels of ROS, generating a damaging state of reductive stress [150]. In vitro scavenging of ROS suppresses tyrosine phosphorylation events associated with sperm capacitation, inhibiting hyperactivated motility, disrupting acrosomal exocytosis, and, in animal models, reducing fertilization rates [49,151,152,153,154]. Similarly, with oocyte maturation, while low doses of antioxidants can facilitate this process, high doses delay or completely suppress the ability of mammalian oocytes to undergo meiotic maturation in vitro and ovulate in vivo [155,156,157,158,159]. High doses of antioxidants have also been found to have a negative impact on both cleavage and blastocyst development rates in vitro, while compounds with antioxidant properties such as sanguinarine and EGCG have also been found to suppress blastocyst implantation and post-implantation embryonic development [160,161]. Later in pregnancy, coenzyme Q10 administration has been found to increase OS in rats [162]. Furthermore, because ROS plays a crucial role in the fetal brain-sparing response (prioritization of oxygen and nutrient delivery to the brain, heart, and adrenal glands at the expense of other organs during pregnancy), there are also concerns that excessive antioxidant use by pregnant women could weaken fetal defences against acute hypoxia, increasing the risk of hypoxic–ischaemic encephalopathy [163]. Towards the end of pregnancy, antioxidants have been used in an attempt to address complications such as pre-eclampsia, pre-term labour, fetal death, fetal growth restriction, and stillbirth, but no positive outcomes have been recorded. Indeed, occasionally detrimental impacts of such treatment have even surfaced, including decreases in human chorionic gonadotrophin generation, fetal growth restriction, low birthweight, gestational hypertension, and others [164,165,166,167].

In terms of mechanisms, over-supplementation with antioxidants can drive mitochondrial ROS generation by enhancing the reduced status of key electron donors to the ETC. This leads to increases in the NADH:NAD^+^ and FADH_2_:FAD ratios to the point that the ETC cannot cope; electrons leak directly, or following reverse electron transport to Complex I, and are swept up by oxygen to generate O_2_^•−^, which then rapidly dismutates to H_2_O_2_ under the influence of SOD. Similarly, increases in the NADPH:NADP^+^ ratio can favour the activation of NADPH oxidases such as NOX 4 and NOX5, generating excess ROS and impairing cell function [168].

High doses of powerful reductants like vitamin C can also enhance OS by promoting Fenton chemistry, whereby the reduced form of transition metals, such as iron and copper, can promote the generation of free radicals. High levels of certain antioxidants can also interfere with cellular homeostasis by disrupting the intricate redox-signalling processes highlighted above or by interfering with critical homeostatic mechanisms such as apoptosis or protein folding. Some antioxidants, such as polyphenols, may also have chemical structures (large planar hydrophobic molecules) that, at high concentrations, can insinuate themselves into membranes, disrupting cellular activity by promoting electron leakage and ROS generation from the mitochondria or disrupting receptor activation and signal transduction at the plasma membrane. They can also intercalate into the DNA, distorting the DNA backbone, disrupting chromatin compaction, and inducing DNA fragmentation [169,170,171]. At high doses, instead of protecting the genome from OS, such molecules can induce DNA strand breaks and/or inhibit repair enzymes like topoisomerase [172].

So, while there is abundant enthusiasm for the use of antioxidant supplements to treat a range of reproductive pathologies, we are still a long way short of this goal. First of all, we lack a simple, clinically validated test to determine which patients are suffering from OS and require antioxidant treatment. The same lack of a diagnostic test means that we do not know when to cease antioxidant treatment, so that the risk of over-supplementation and the instigation of reductive stress can be avoided [173,174]. The specific antioxidants that should be used, in terms of their bioavailability, mechanism of action, and site of action, have still not been optimized for different clinical conditions, and the quality of clinical trials in this area has been generally poor [175]. The indiscriminate use of poorly selected antioxidants to treat reproductive disorders, without regard for the redox status of the patient, the source, and biochemical nature of the stress, or the danger of over-supplementation, has led to widespread concern that this treatment strategy is ineffective. According to the WHO, “There are insufficient data to recommend the use of supplemental antioxidant therapies for the treatment of men with abnormal semen parameters and/or male infertility” [176]. Similarly, antioxidants have yielded disappointing results when used to manage complications of pregnancy [164], endometriosis [177], polycystic ovarian syndrome (PCOS) [178], and female infertility [179]. Although there are signs of definite promise for these reagents, much more fundamental work needs to be done on the diagnosis and characterization of OS before antioxidant supplementation can be adopted as an effective, robust, therapeutic option.

## 9. Conclusions

This review clearly emphasises that ROS is physiologically important at all stages of the reproductive process. While OS may well contribute towards many of the pathologies affecting human fertility, antioxidants should be utilised with care, with the aim of ensuring that an appropriate redox balance is maintained and that critical biological processes can continue unimpaired. This is especially important as there is now growing evidence for reductive stress negatively impacting the reproductive process from gametogenesis to parturition [173]. To rationalize the use of antioxidants to treat reproductive deficiencies in vivo and in vitro, we need to consider much more carefully the dose and specific structure of the antioxidants used in clinical practice. Too often, antioxidant administration is not calibrated with the level of OS being experienced by the patient or the cells being targeted. Indeed, antioxidants are frequently administered in vivo and in vitro without any diagnostic assessment of OS [175]. In addition, too little consideration has been given to the source and type of OS when selecting antioxidants in terms of their physicochemical properties (charge, size, hydrophobicity, half-life, bioavailabilty) or mode of antioxidant action (one electron-, two electron-, or hydrogen atom-donating) in optimizing their biological action. With the introduction of new ART culture media formulations enriched with antioxidants, and an abundance of articles highlighting the negative impacts of ROS on ART outcomes, there is a risk that antioxidants will be used in doses that compromise the physiological role of ROS in driving the reproductive process and inadvertently create a state of reductive stress, which can be just as damaging as its oxidative counterpart.

## Data Availability

No new data were created or analyzed in this study.

## References

[1] Mostafa T., Anis T., El Nashar A., Imam H., Osman I. (2012). Seminal plasma reactive oxygen species–antioxidants relationship with varicocele grade. Andrologia.

[2] Fang Y., Su Y., Xu J., Hu Z., Zhao K., Liu C., Zhang H. (2021). Varicocele-mediated male infertility: From the perspective of testicular immunity and inflammation. Front. Immunol..

[3] Liu Y., Yu Z., Zhao S., Cheng L., Man Y., Gao X., Zhao H. (2021). Oxidative stress markers in the follicular fluid of patients with polycystic ovary syndrome correlate with a decrease in embryo quality. J. Assist. Reprod. Genet..

[4] Kumari S., Dcunha R., Sanghvi S.P., Nayak G., Kalthur S.G., Raut S.Y., Mutalik S., Siddiqui S., Alrumman S.A., Adiga S.K. (2021). Organophosphorus pesticide quinalphos (Ekalux 25 E.C.) reduces sperm functional competence and decreases the fertilisation potential in Swiss albino mice. Andrologia.

[5] Mohammadi-Sardoo M., Mandegary A., Nabiuni M., Nematollahi-Mahani S.N., Amirheidari B. (2018). Mancozeb induces testicular dysfunction through oxidative stress and apoptosis: Protective role of N-acetylcysteine antioxidant. Toxicol. Ind. Health.

[6] De Iuliis G.N., Newey R.J., King B.V., Aitken R.J. (2009). Mobile phone radiation induces reactive oxygen species production and DNA damage in human spermatozoa in vitro. PLoS ONE.

[7] Juan-Reyes S.S., Gómez-Oliván L.M., Juan-Reyes N.S., Islas-Flores H., Dublán-García O., Orozco-Hernández J.M., Pérez-Álvarez I., Mejía-García A. (2023). Women with preeclampsia exposed to air pollution during pregnancy: Relationship between oxidative stress and neonatal disease—Pilot study. Sci. Total Environ..

[8] Abbasihormozi S.H., Babapour V., Kouhkan A., Niasari Naslji A., Afraz K., Zolfaghary Z., Shahverdi A.H. (2019). Stress hormone and oxidative stress biomarkers link obesity and diabetes with reduced fertility potential. Cell J..

[9] DeMarini D.M. (2004). Genotoxicity of tobacco smoke and tobacco smoke condensate: A review. Mutat. Res..

[10] Ferramosca A., Conte A., Zara V. (2015). Krill oil ameliorates mitochondrial dysfunctions in rats treated with high-fat diet. Biomed Res. Int..

[11] Ballesteros-Guzmán A.K., Carrasco-Legleu C.E., Levario-Carrillo M., Chávez-Corral D.V., Sánchez-Ramírez B., Mariñelarena-Carrillo E.O., Guerrero-Salgado F., Reza-López S.A. (2019). Prepregnancy obesity, maternal dietary intake, and oxidative stress biomarkers in the fetomaternal unit. Biomed Res. Int..

[12] Aitken R.J. (2022). Male infertility and oxidative stress: A focus on the underlying mechanisms. Antioxidants.

[13] Sies H. (2015). Oxidative stress: A concept in redox biology and medicine. Redox Biol..

[14] Kamkar N., Ramezanali F., Sabbaghian M. (2018). The relationship between sperm DNA fragmentation, free radicals, and antioxidant capacity with idiopathic repeated pregnancy loss. Reprod. Biol..

[15] Hadi T., Bardou M., Mace G., Sicard P., Wendremaire M., Barrichon M., Richaud S., Demidov O., Sagot P., Garrido C. (2015). Glutathione prevents preterm parturition and fetal death by targeting macrophage-induced reactive oxygen species production in the myometrium. FASEB J..

[16] Puttabyatappa M., Jacot T.A., Al-Alem L.F., Rosewell K.L., Duffy D.M., Brännström M., Curry T.E. (2014). Ovarian membrane-type matrix metalloproteinases: Induction of MMP14 and MMP16 during the periovulatory period in the rat, macaque, and human. Biol. Reprod..

[17] Kim D.J., Iwasaki A., Chien A.L., Kang S. (2022). UVB-mediated DNA damage induces matrix metalloproteinases to promote photoaging in an AhR- and SP1-dependent manner. JCI Insight.

[18] Kalous J., Aleshkina D., Anger M. (2023). A role of PI3K/Akt signaling in oocyte maturation and early embryo development. Cells.

[19] Koppers A.J., Mitchell L.A., Wang P., Lin M., Aitken R.J. (2011). Phosphoinositide 3-kinase signalling pathway involvement in a truncated apoptotic cascade associated with motility loss and oxidative DNA damage in human spermatozoa. Biochem. J..

[20] Zhao H., Dinh T.H., Wang Y., Yang Y. (2025). The roles of MAPK signaling pathway in ovarian folliculogenesis. J. Ovarian Res..

[21] Luo D., He Z., Yu C., Guan Q. (2022). Role of p38 MAPK signalling in testis development and male fertility. Oxid. Med. Cell Longev..

[22] Signorini C., Saso L., Ghareghomi S., Telkoparan-Akıllılar P., Collodel G., Moretti E. (2024). Redox homeostasis and Nrf2-regulated mechanisms are relevant to male infertility. Antioxidants.

[23] Chapple S.J., Puszyk W.M., Mann G.E. (2015). Keap1–Nrf2 regulated redox signaling in utero: Priming of disease susceptibility in offspring. Free Radic. Biol. Med..

[24] Kabe Y., Ando K., Hirao S., Yoshida M., Handa H. (2005). Redox regulation of NF-κB activation: Distinct redox regulation between the cytoplasm and the nucleus. Antioxid. Redox Signal..

[25] Aitken R.J., Gibb Z., Baker M.A., Drevet J., Gharagozloo P. (2016). Causes and consequences of oxidative stress in spermatozoa. Reprod. Fertil. Dev..

[26] Liu M., Wu K., Wu Y. (2023). The emerging role of ferroptosis in female reproductive disorders. Biomed. Pharmacother..

[27] Eghtedari A.R., Safizadeh B., Vaezi M.A., Kalantari S., Tavakoli-Yaraki M. (2022). Functional and pathological role of 15-lipoxygenase and its metabolites in pregnancy and pregnancy-associated complications. Prostaglandins Other Lipid Mediat..

[28] Mruk D.D., Silvestrini B., Mo M.Y., Cheng C.Y. (2002). Antioxidant superoxide dismutase—A review: Its function, regulation in the testis, and role in male fertility. Contraception.

[29] Herman S., Lipiński P., Ogórek M., Starzyński R., Grzmil P., Bednarz A., Lenartowicz M. (2020). Molecular regulation of copper homeostasis in the male gonad during the process of spermatogenesis. Int. J. Mol. Sci..

[30] Onochie C., Evi K., O’Flaherty C. (2025). Role of redox-induced protein modifications in spermatozoa in health and disease. Antioxidants.

[31] Ursini F., Heim S., Kiess M., Maiorino M., Roveri A., Wissing J., Flohé L. (1999). Dual function of the selenoprotein PHGPx during sperm maturation. Science.

[32] Aitken R.J., De Iuliis G.N., Nixon B. (2020). The sins of our forefathers: Paternal impacts on de novo mutation rate and development. Annu. Rev. Genet..

[33] Ribas-Maynou J., Nguyen H., Wu H., Ward W.S. (2022). Functional aspects of sperm chromatin organization. Results Probl. Cell Differ..

[34] Chabory E., Damon C., Lenoir A., Henry-Berger J., Vernet P., Cadet R., Saez F., Drevet J.R. (2010). Mammalian glutathione peroxidases control acquisition and maintenance of spermatozoa integrity. J. Anim. Sci..

[35] Pfeifer H., Conrad M., Roethlein D., Kyriakopoulos A., Brielmeier M., Bornkamm G.W., Behne D. (2001). Identification of a specific sperm nuclei selenoenzyme necessary for protamine thiol cross-linking during sperm maturation. FASEB J..

[36] Bennetts L.E., Aitken R.J. (2005). A comparative study of oxidative DNA damage in mammalian spermatozoa. Mol. Reprod. Dev..

[37] Ward W.S. (2018). Organization of sperm DNA by the nuclear matrix. Am. J. Clin. Exp. Urol..

[38] Bianchi P.G., Manicardi G.C., Bizzaro D., Bianchi U., Sakkas D. (1993). Effect of deoxyribonucleic acid protamination on fluorochrome staining and in situ nick-translation of murine and human mature spermatozoa. Biol. Reprod..

[39] Oliva R. (2006). Protamines and male infertility. Hum. Reprod. Update.

[40] Zini A., Kamal K.M., Phang D. (2001). Free thiols in human spermatozoa: Correlation with sperm DNA integrity. Urology.

[41] Aitken R.J., Curry B.J. (2011). Redox regulation of human sperm function: From the physiological control of sperm capacitation to the etiology of infertility and DNA damage in the germ line. Antioxid. Redox Signal..

[42] Welsh C.L., Madan L.K. (2024). Protein tyrosine phosphatase regulation by reactive oxygen species. Adv. Cancer Res..

[43] Aitken R.J., Harkiss D., Knox W., Paterson M., Irvine D.S. (1998). A novel signal transduction cascade in capacitating human spermatozoa characterised by a redox-regulated, cAMP-mediated induction of tyrosine phosphorylation. J. Cell Sci..

[44] Zhang H., Zheng R.L. (1996). Promotion of human sperm capacitation by superoxide anion. Free Radic. Res..

[45] Rivlin J., Mendel J., Rubinstein S., Etkovitz N., Breitbart H. (2004). Role of hydrogen peroxide in sperm capacitation and acrosome reaction. Biol. Reprod..

[46] Boerke A., Brouwers J.F., Olkkonen V.M., van de Lest C.H., Sostaric E., Schoevers E.J., Helms J.B., Gadella B.M. (2013). Involvement of bicarbonate-induced radical signaling in oxysterol formation and sterol depletion of capacitating mammalian sperm during in vitro fertilization. Biol. Reprod..

[47] Griveau J.F., Renard P., Le Lannou D. (1995). Superoxide anion production by human spermatozoa as a part of the ionophore-induced acrosome reaction process. Int. J. Androl..

[48] de Lamirande E., Gagnon C. (1993). A positive role for the superoxide anion in triggering hyperactivation and capacitation of human spermatozoa. Int. J. Androl..

[49] Aitken R.J., Paterson M., Fisher H., Buckingham D.W., van Duin M. (1995). Redox regulation of tyrosine phosphorylation in human spermatozoa and its role in the control of human sperm function. J. Cell Sci..

[50] Serafini S., O’Flaherty C. (2022). Redox regulation to modulate phosphorylation events in human spermatozoa. Antioxid. Redox Signal.

[51] Voldborg B.R., Damstrup L., Spang-Thomsen M., Poulsen H.S. (1997). Epidermal growth factor receptor (EGFR) and EGFR mutations: Function and possible role in clinical trials. Ann. Oncol..

[52] León-Buitimea A., Rodríguez-Fragoso L., Lauer F.T., Bowles H., Thompson T.A., Burchiel S.W. (2012). Ethanol-induced oxidative stress is associated with EGF receptor phosphorylation in MCF-10A cells overexpressing CYP2E1. Toxicol. Lett..

[53] Papaiahgari S., Zhang Q., Kleeberger S.R., Cho H.Y., Reddy S.P. (2006). Hyperoxia stimulates an Nrf2-ARE transcriptional response via ROS-EGFR-PI3K-Akt/ERK MAP kinase signaling in pulmonary epithelial cells. Antioxid. Redox Signal..

[54] Shahar S., Hillman P., Lubart R., Ickowicz D., Breitbart H. (2014). Activation of sperm EGFR by light irradiation is mediated by reactive oxygen species. Photochem. Photobiol..

[55] Finkelstein M., Etkovitz N., Breitbart H. (2020). Ca^2+^ signaling in mammalian spermatozoa. Mol. Cell Endocrinol..

[56] Aitken R.J. (2020). The importance of oxidative stress in determining the functionality of mammalian spermatozoa: A two-edged sword. Antioxidants.

[57] Redgrove K.A., Anderson A.L., McLaughlin E.A., O’Bryan M.K., Aitken R.J., Nixon B. (2013). Investigation of the mechanisms by which the molecular chaperone HSPA2 regulates the expression of sperm surface receptors involved in human sperm–oocyte recognition. Mol. Hum. Reprod..

[58] Llanos M.N., Morales P., Riffo M.S. (1993). Studies of lysophospholipids related to the hamster sperm acrosome reaction in vitro. J. Exp. Zool..

[59] Goldman R., Ferber E., Zort U. (1992). Reactive oxygen species are involved in the activation of cellular phospholipase A2. FEBS Lett..

[60] Aitken R.J., Gordon E., Harkiss D., Twigg J.P., Milne P., Jennings Z., Irvine D.S. (1998). Relative impact of oxidative stress on the functional competence and genomic integrity of human spermatozoa. Biol. Reprod..

[61] Zmijewski J.W., Banerjee S., Bae H., Friggeri A., Lazarowski E.R., Abraham E. (2010). Exposure to hydrogen peroxide induces oxidation and activation of AMP-activated protein kinase. J. Biol. Chem..

[62] Calle-Guisado V., de Llera A.H., Martin-Hidalgo D., Mijares J., Gil M.C., Alvarez I.S., Bragado M.J., Garcia-Marin L.J. (2017). AMP-activated kinase in human spermatozoa: Identification, intracellular localization, and key function in the regulation of sperm motility. Asian J. Androl..

[63] Aitken R.J., De Iuliis G.N. (2010). On the possible origins of DNA damage in human spermatozoa. Mol. Hum. Reprod..

[64] Aitken R.J., Baker M.A., Nixon B. (2015). Are sperm capacitation and apoptosis the opposite ends of a continuum driven by oxidative stress?. Asian J. Androl..

[65] O’Flaherty C. (2025). Redox signaling regulation in human spermatozoa: A primary role of peroxiredoxins. Asian J. Androl..

[66] Voros C., Athanasiou D., Papapanagiotou I., Mavrogianni D., Varthaliti A., Bananis K., Athanasiou A., Athanasiou A., Papadimas G., Gkirgkinoudis A. (2025). Cracking the code of oocyte quality: The oxidative stress link to IVF success. Int. J. Mol. Sci..

[67] Rodríguez-Nuevo A., Torres-Sanchez A., Duran J.M., De Guirior C., Martínez-Zamora M.A., Böke E. (2022). Oocytes maintain ROS-free mitochondrial metabolism by suppressing complex I. Nature.

[68] Bahety D., Böke E., Rodríguez-Nuevo A. (2024). Mitochondrial morphology, distribution and activity during oocyte development. Trends Endocrinol. Metab..

[69] Chen Q., Zhang W., Ran H., Feng L., Yan H., Mu X., Han Y., Liu W., Xia G., Wang C. (2014). PKCδ and θ possibly mediate FSH-induced mouse oocyte maturation via NOX-ROS-TACE cascade signaling pathway. PLoS ONE.

[70] von Mengden L., Klamt F., Smitz J. (2020). Redox biology of human cumulus cells: Basic concepts, impact on oocyte quality, and potential clinical use. Antioxid. Redox Signal..

[71] Kala M., Shaikh M.V., Nivsarkar M. (2016). Equilibrium between antioxidants and reactive oxygen species: A requisite for oocyte development and maturation. Reprod. Med. Biol..

[72] Chen J., Hudson E., Chi M.M., Chang A.S., Moley K.H., Hardie D.G., Downs S.M. (2006). AMPK regulation of mouse oocyte meiotic resumption in vitro. Dev. Biol..

[73] Guarnaccia M.M., Takami M., Jones E.E., Preston S.L., Behrman H.R. (2000). Luteinizing hormone depletes ascorbic acid in preovulatory follicles. Fertil. Steril..

[74] Takami M., Preston S.L., Toyloy V.A., Behrman H.R. (1999). Antioxidants reversibly inhibit the spontaneous resumption of meiosis. Am. J. Physiol..

[75] Shkolnik K., Tadmor A., Ben-Dor S., Nevo N., Galiani D., Dekel N. (2011). Reactive oxygen species are indispensable in ovulation. Proc. Natl. Acad. Sci. USA.

[76] Kawashima I., Liu Z., Mullany L.K., Mihara T., Richards J.S., Shimada M. (2012). EGF-like factors induce expansion of the cumulus cell–oocyte complexes by activating calpain-mediated cell movement. Endocrinology.

[77] Begum I.A. (2025). Oxidative stress: Oocyte quality and infertility. Reprod. Toxicol..

[78] Hennet M.L., Yu H.Y., Combelles C.M. (2013). Follicular fluid hydrogen peroxide and lipid hydroperoxide in bovine antral follicles of various size, atresia, and dominance status. J. Assist. Reprod. Genet..

[79] Liu S., Jia Y., Meng S., Luo Y., Yang Q., Pan Z. (2023). Mechanisms of and potential medications for oxidative stress in ovarian granulosa cells: A review. Int. J. Mol. Sci..

[80] Wang S., He G., Chen M., Zuo T., Xu W., Liu X. (2017). The role of antioxidant enzymes in the ovaries. Oxid. Med. Cell Longev..

[81] Varga D., Szatmári P., Ducza E. (2025). Inflammatory and redox mediators in rat and human ovulation. Int. J. Mol. Sci..

[82] Brännström M., Bonello N., Norman R.J., Robertson S.A. (1995). Reduction of ovulation rate in the rat by administration of a neutrophil-depleting monoclonal antibody. J. Reprod. Immunol..

[83] Richards J.S., Russell D.L., Ochsner S., Espey L.L. (2002). Ovulation: New dimensions and new regulators of the inflammatory-like response. Annu. Rev. Physiol..

[84] Russell D.L., Robker R.L. (2007). Molecular mechanisms of ovulation: Co-ordination through the cumulus complex. Hum. Reprod. Update.

[85] Shrestha K., Puttabyatappa M., Wynn M.A., Hannon P.R., Al-Alem L.F., Rosewell K.L., Akin J., Curry T.E. (2024). Protease expression in the human and rat cumulus-oocyte complex during the periovulatory period: A role in cumulus-oocyte complex migration. Biol. Reprod..

[86] Richani D., Gilchrist R.B. (2018). The epidermal growth factor network: Role in oocyte growth, maturation, and developmental competence. Hum. Reprod. Update.

[87] Kampfer C., Saller S., Windschüttl S., Berg D., Berg U., Mayerhofer A. (2014). Pigment-epithelium-derived factor (PEDF) and the human ovary: A role in the generation of ROS in granulosa cells. Life Sci..

[88] Lin N., van Zomeren K.C., Plosch T., Hofsink N., van Veen T., Li H.T., Lin J., Zhou X., Groen H., Tietge U.J.F. (2025). Follicle-stimulating hormone stimulates free radical generation without inducing substantial oxidative stress in human granulosa cells. Hum. Reprod. Open.

[89] Puddu P., Puddu G.M., Cravero E., Rosati M., Muscari A. (2008). The molecular sources of reactive oxygen species in hypertension. Blood Press..

[90] Duffy D.M., Ko C., Jo M., Brannstrom M., Curry T.E. (2019). Ovulation: Parallels with inflammatory processes. Endocr. Rev..

[91] Li W., Young J.F., Sun J. (2018). NADPH oxidase-generated reactive oxygen species in mature follicles are essential for *Drosophila* ovulation. Proc. Natl. Acad. Sci. USA.

[92] Miyazaki T., Sueoka K., Dharmarajan A.M., Atlas S.J., Bulkley G.B., Wallach E.E. (1991). Effect of inhibition of oxygen free radical on ovulation and progesterone production by the in vitro perfused rabbit ovary. J. Reprod. Fertil..

[93] Ramalho-Santos J., Amaral S. (2013). Mitochondria and mammalian reproduction. Mol. Cell Endocrinol..

[94] Yalu R., Oyesiji A.E., Eisenberg I., Imbar T., Meidan R. (2015). HIF1A-dependent increase in endothelin 2 levels in granulosa cells: Role of hypoxia, LH/cAMP, and reactive oxygen species. Reproduction.

[95] Behrman H.R., Kodaman P.H., Preston S.L., Gao S. (2001). Oxidative stress and the ovary. J. Soc. Gynecol. Investig..

[96] Tang Z., Chen J., Zhang Z., Bi J., Xu R., Lin Q., Wang Z. (2021). HIF-1α activation promotes luteolysis by enhancing ROS levels in the corpus luteum of pseudopregnant rats. Oxid. Med. Cell Longev..

[97] Luo W., Salih S.M., Bormann C.L., Wiltbank M.C. (2015). Induction of chemokines and prostaglandin synthesis pathways in luteinized human granulosa cells: Potential role of luteotropin withdrawal and prostaglandin F2α in regression of the human corpus luteum. Reprod. Biol..

[98] Evans J., Salamonsen L.A. (2012). Inflammation, leukocytes, and menstruation. Rev. Endocr. Metab. Disord..

[99] Wu B., Chen X., He B., Liu S., Li Y., Wang Q., Gao H., Wang S., Liu J., Zhang S. (2014). ROS are critical for endometrial breakdown via NF-κB–COX-2 signaling in a female mouse menstrual-like model. Endocrinology.

[100] Lord T., Martin J.H., Aitken R.J. (2015). Accumulation of electrophilic aldehydes during postovulatory aging of mouse oocytes causes reduced fertility, oxidative stress, and apoptosis. Biol. Reprod..

[101] Gardner D.K., Pool T.B., Lane M. (2000). Embryo nutrition and energy metabolism and its relationship to embryo growth, differentiation, and viability. Semin. Reprod. Med..

[102] Houghton F.D., Thompson J.G., Kennedy C.J., Leese H.J. (1996). Oxygen consumption and energy metabolism of the early mouse embryo. Mol. Reprod. Dev..

[103] Kimura N., Tsunoda S., Iuchi Y., Abe H., Totsukawa K., Fujii J. (2010). Intrinsic oxidative stress causes either 2-cell arrest or cell death depending on developmental stage of the embryos from SOD1-deficient mice. Mol. Hum. Reprod..

[104] Nasr-Esfahani M.H., Aitken R.J., Johnson M.H. (1990). Hydrogen peroxide levels in mouse oocytes and early cleavage stage embryos developed in vitro or in vivo. Development.

[105] Legge M., Sellens M.H. (1991). Free radical scavengers ameliorate the 2-cell block in mouse embryo culture. Hum. Reprod..

[106] Mu X.F., Jin X.L., Farnham M.M., Li Y., O’Neill C. (2011). DNA damage-sensing kinases mediate the mouse 2-cell embryo’s response to genotoxic stress. Biol. Reprod..

[107] Harvey A.J. (2007). The role of oxygen in ruminant preimplantation embryo development and metabolism. Anim. Reprod. Sci..

[108] Jurisicova A., Acton B.M. (2004). Deadly decisions: The role of genes regulating programmed cell death in human preimplantation embryo development. Reproduction..

[109] Son Y., Cheong Y.K., Kim N.H., Chung H.T., Kang D.G., Pae H.O. (2011). Mitogen-activated protein kinases and reactive oxygen species: How can ROS activate MAPK pathways?. J. Signal Transduct..

[110] Tang C., Liang J., Qian J., Jin L., Du M., Li M., Li D. (2014). Opposing role of JNK-p38 kinase and ERK1/2 in hydrogen peroxide-induced oxidative damage of human trophoblast-like JEG-3 cells. Int. J. Clin. Exp. Pathol..

[111] Ji A.R., Ku S.Y., Cho M.S., Kim Y.Y., Kim Y.J., Oh S.K., Kim S.H., Moon S.Y., Choi Y.M. (2010). Reactive oxygen species enhance differentiation of human embryonic stem cells into mesendodermal lineage. Exp. Mol. Med..

[112] Fourquet S., Guerois R., Biard D., Toledano M.B. (2010). Activation of NRF2 by nitrosative agents and H_2_O_2_ involves KEAP1 disulfide formation. J. Biol. Chem..

[113] Hardy M.L.M., Day M.L., Morris M.B. (2021). Redox regulation and oxidative stress in mammalian oocytes and embryos developed in vivo and in vitro. Int. J. Environ. Res. Public Health.

[114] Deluao J.C., Winstanley Y., Robker R.L., Pacella-Ince L., Gonzalez M.B., McPherson N.O. (2022). Oxidative stress and reproductive function: Reactive oxygen species in the mammalian pre-implantation embryo. Reproduction.

[115] Li L. (2017). The relevance of mammalian peroxiredoxins to the gametogenesis, embryogenesis, and pregnancy outcomes. Reprod. Sci..

[116] Guérin P., El Mouatassim S., Ménézo Y. (2001). Oxidative stress and protection against reactive oxygen species in the pre-implantation embryo and its surroundings. Hum. Reprod. Update.

[117] Laloraya M., Kumar G.P., Laloraya M.M. (1989). A possible role of superoxide anion radical in the process of blastocyst implantation in *Mus. musculus*. Biochem. Biophys. Res. Commun..

[118] Sunuwar S., Heo Y.S. (2026). Reactive oxygen species in embryo development: Sources, impacts, and implications for in vitro culture systems. Life.

[119] Thomas M., Jain S., Kumar G.P., Laloraya M. (1997). A programmed oxyradical burst causes hatching of mouse blastocysts. J. Cell Sci..

[120] Manes C., Lai N.C. (1995). Nonmitochondrial oxygen utilization by rabbit blastocysts and surface production of superoxide radicals. J. Reprod. Fertil..

[121] Mishra A., Seshagiri P.B. (2000). Heparin-binding epidermal growth factor improves blastocyst hatching and trophoblast outgrowth in the golden hamster. Reprod. Biomed. Online.

[122] Seshagiri P.B., Roy S.S., Sireesha G., Rao R.P. (2009). Cellular and molecular regulation of mammalian blastocyst hatching. J. Reprod. Immunol..

[123] Kim S.M., Kim J.S. (2017). A review of mechanisms of implantation. Dev. Reprod..

[124] Geraldo L.H.M., Spohr T.C.L.S., Amaral R.F.D., Fonseca A.C.C.D., Garcia C., Mendes F.A., Freitas C., dosSantos M.F., Lima F.R.S. (2021). Role of lysophosphatidic acid and its receptors in health and disease: Novel therapeutic strategies. Signal Transduct. Target. Ther..

[125] Fritz R., Jain C., Armant D.R. (2014). Cell signaling in trophoblast-uterine communication. Int. J. Dev. Biol..

[126] Beltrame J.S., Sordelli M.S., Cella M., Perez Martinez S., Franchi A.M., Ribeiro M.L. (2013). Lysophosphatidic acid increases the production of pivotal mediators of decidualization and vascularization in the rat uterus. Placenta.

[127] Okada H., Tsuzuki T., Murata H. (2018). Decidualization of the human endometrium. Reprod. Med. Biol..

[128] Dunn C.L., Kelly R.W., Critchley H.O. (2003). Decidualization of the human endometrial stromal cell: An enigmatic transformation. Reprod. Biomed. Online.

[129] Gellersen B., Brosens J. (2003). Cyclic AMP and progesterone receptor cross-talk in human endometrium: A decidualizing affair. J. Endocrinol..

[130] Al-Sabbagh M., Fusi L., Higham J., Lee Y., Lei K., Hanyaloglu A.C., Lam E.W., Christian M., Brosens J.J. (2011). NADPH oxidase-derived reactive oxygen species mediate decidualization of human endometrial stromal cells in response to cyclic AMP signaling. Endocrinology.

[131] Salker M.S., Christian M., Steel J.H., Nautiyal J., Lavery S., Trew G., Webster Z., Al-Sabbagh M., Puchchakayala G., Föller M. (2011). Deregulation of the serum- and glucocorticoid-inducible kinase SGK1 in the endometrium causes reproductive failure. Nat. Med..

[132] Lou Y., Hu M., Mao L., Zheng Y., Jin F. (2017). Involvement of serum glucocorticoid-regulated kinase 1 in reproductive success. FASEB J..

[133] Sugino N. (2007). The role of oxygen radical-mediated signaling pathways in endometrial function. Placenta.

[134] Mary S., Kulkarni M.J., Mehendale S.S., Joshi S.R., Giri A.P. (2017). Tubulointerstitial nephritis antigen-like 1 protein is downregulated in the placenta of pre-eclamptic women. Clin. Proteom..

[135] Matsumoto H. (2017). Molecular and cellular events during blastocyst implantation in the receptive uterus: Clues from mouse models. J. Reprod. Dev..

[136] Varisli L., Tolan V. (2022). Increased ROS alters E-/N-cadherin levels and promotes migration in prostate cancer cells. Bratisl. Lek. Listy.

[137] Zhao H.B., Wang C., Li R.X., Tang C.L., Li M.Q., Du M.R., Hou X.F., Li D.J. (2010). E-cadherin, as a negative regulator of invasive behavior of human trophoblast cells, is down-regulated by cyclosporin A via epidermal growth factor/extracellular signal-regulated protein kinase signaling pathway. Biol. Reprod..

[138] Wang Y., Ma J., Shen H., Wang C., Sun Y., Howell S.B., Lin X. (2014). Reactive oxygen species promote ovarian cancer progression via the HIF-1α/LOX/E-cadherin pathway. Oncol. Rep..

[139] Brüne B., Zhou J. (2003). The role of nitric oxide (NO) in stability regulation of hypoxia inducible factor-1α (HIF-1α). Curr. Med. Chem..

[140] Lyall F. (2003). Development of the utero-placental circulation: The role of carbon monoxide and nitric oxide in trophoblast invasion and spiral artery transformation. Microsc. Res. Tech..

[141] Huppertz B., Kertschanska S., Demir A.Y., Frank H.G., Kaufmann P. (1998). Immunohistochemistry of matrix metalloproteinases (MMP), their substrates, and their inhibitors (TIMP) during trophoblast invasion in the human placenta. Cell Tissue Res..

[142] Nawrocki B., Polette M., Maquoi E., Birembaut P. (1997). Expression of matrix metalloproteinases and their inhibitors during human placental development. Placenta.

[143] Sánchez-Santos A., Martínez-Hernández M.G., Contreras-Ramos A., Ortega-Camarillo C., Baiza-Gutman L.A. (2018). Hyperglycemia-induced mouse trophoblast spreading is mediated by reactive oxygen species. Mol. Reprod. Dev..

[144] Li L., Zhou Y., Zhou W., Liu Y., Mei J. (2025). Fibroblast growth factor 7 facilitates invasion of human trophoblast cells through the JNK pathway during pregnancy. Placenta.

[145] Zhan X., Xie Y., Sun L., Si Q., Shang H. (2021). Dexamethasone may inhibit placental growth by blocking glucocorticoid receptors via phosphatidylinositol 3-kinase/AKT/mammalian target of rapamycin and reactive oxygen species/AMP-activated protein kinase signalling pathways in human placental JEG-3 cells. Reprod. Fertil. Dev..

[146] Sheller-Miller S., Richardson L., Martin L., Jin J., Menon R. (2018). Systematic review of p38 mitogen-activated kinase and its functional role in reproductive tissues. Am. J. Reprod. Immunol..

[147] Kim S.H., MacIntyre D.A., Firmino Da Silva M., Blanks A.M., Lee Y.S., Thornton S., Bennett P.R., Terzidou V. (2015). Oxytocin activates NF-κB-mediated inflammatory pathways in human gestational tissues. Mol. Cell Endocrinol..

[148] Mandalà M. (2025). Oxidative stress and inflammation in uterine-vascular adaptation during pregnancy. Antioxidants.

[149] Chang E.Y., Zhang J., Sullivan S., Newman R., Singh I. (2012). N-acetylcysteine prevents preterm birth by attenuating the LPS-induced expression of contractile associated proteins in an animal model. J. Matern. Fetal Neonatal Med..

[150] Sadeghi N., Boissonneault G., Tavalaee M., Nasr-Esfahani M.H. (2023). Oxidative versus reductive stress: A delicate balance for sperm integrity. Syst. Biol. Reprod. Med..

[151] Leclerc P., de Lamirande E., Gagnon C. (1997). Regulation of protein-tyrosine phosphorylation and human sperm capacitation by reactive oxygen derivatives. Free Radic. Biol. Med..

[152] de Lamirande E., Tsai C., Harakat A., Gagnon C. (1998). Involvement of reactive oxygen species in human sperm acrosome reaction induced by A23187, lysophosphatidylcholine, and biological fluid ultrafiltrates. J. Androl..

[153] Sapanidou V., Taitzoglou I., Tsakmakidis Ι., Kourtzelis I., Fletouris D., Theodoridis A., Zervos I., Tsantarliotou M. (2015). Antioxidant effect of crocin on bovine sperm quality and in vitro fertilization. Theriogenology.

[154] Takei G.L., Tourzani D.A., Paudel B., Visconti P.E. (2021). Activation of cAMP-dependent phosphorylation pathways is independent of ROS production during mouse sperm capacitation. Mol. Reprod. Dev..

[155] Park Y.G., Lee S.E., Son Y.J., Jeong S.G., Shin M.Y., Kim W.J., Kim E.Y., Park S.P. (2018). Antioxidant β-cryptoxanthin enhances porcine oocyte maturation and subsequent embryo development in vitro. Reprod. Fertil. Dev..

[156] Wei P., Wang J., Yu H., Chen Y., Liu C., Zhang Y., Zeng W., Hu G. (2024). Effects of leonurine on oocyte maturation and parthenogenetic embryo development in sheep. Reprod. Domest. Anim..

[157] Morais A.N.P., Lima L.F., Silva A.F.B., Lienou L.L., Ferreira A.C.A., Watanabe Y.F., Joaquim D.C., Alves B.G., Pereira A.F., Alves D.R. (2023). Effect of carvacrol antioxidant capacity on oocyte maturation and embryo production in cattle. Zygote.

[158] Musapoor S., Davoodian N., Kadivar A., Ahmadi E., Nazari H., Mehrban H. (2023). Gamma-oryzanol dose optimization in maturation or culture media for in vitro ovine oocyte and embryo development. Iran. J. Vet. Res..

[159] Tonekam K., Anthakat Y., Polrachom A., Samruan W., Anwised P., Boonchuen P., Ketudat-Cairns M., Parnpai R. (2025). Resveratrol supplementation in vitro maturation and culture medium: Enhancing blastocyst viability after vitrification. Anim. Sci. J..

[160] Fan Y.C., Chan W.H. (2014). Epigallocatechin gallate induces embryonic toxicity in mouse blastocysts through apoptosis. Drug Chem. Toxicol..

[161] Chan W.H. (2011). Embryonic toxicity of sanguinarine through apoptotic processes in mouse blastocysts. Toxicol. Lett..

[162] Staicu M.L., Mureşan A., Tache S., Moldovan R. (2011). Effects of exogenous antioxidants on oxidative stress in pregnancy. J. Med. Life.

[163] Botting-Lawford K.J., Skeffington K.L., Murphy M.P., David A.L., Giussani D.A. (2025). Antioxidants: Powering the fight against fetal hypoxia. Philos. Trans. R. Soc. Lond. B Biol. Sci..

[164] Di Fabrizio C., Giorgione V., Khalil A., Murdoch C.E. (2022). Antioxidants in pregnancy: Do we really need more trials?. Antioxidants.

[165] Poston L., Igosheva N., Mistry H.D., Seed P.T., Shennan A.H., Rana S., Karumanchi S.A., Chappell L.C. (2011). Role of oxidative stress and antioxidant supplementation in pregnancy disorders. Am. J. Clin. Nutr..

[166] Aris A., Leblanc S., Ouellet A., Moutquin J.M. (2008). Detrimental effects of high levels of antioxidant vitamins C and E on placental function: Considerations for the vitamins in preeclampsia (VIP) trial. J. Obstet. Gynaecol. Res..

[167] Shennan A.H., Duckworth S. (2010). Use of vitamin C and E to prevent preeclampsia. Obstet. Med..

[168] Korge P., Calmettes G., Weiss J.N. (2015). Increased reactive oxygen species production during reductive stress: The roles of mitochondrial glutathione and thioredoxin reductases. Biochim. Biophys. Acta.

[169] Menezo Y., Evenson D., Cohen M., Dale B. (2014). Effect of antioxidants on sperm genetic damage. Adv. Exp. Med. Biol..

[170] Menezo Y.J., Silvestris E., Dale B., Elder K. (2016). Oxidative stress and alterations in DNA methylation: Two sides of the same coin in reproduction. Reprod. Biomed. Online.

[171] Ménézo Y.J., Hazout A., Panteix G., Robert F., Rollet J., Cohen-Bacrie P., Chapuis F., Clément P., Benkhalifa M. (2007). Antioxidants to reduce sperm DNA fragmentation: An unexpected adverse effect. Reprod. Biomed. Online.

[172] Bandele O.J., Osheroff N. (2008). -Epigallocatechin gallate, a major constituent of green tea, poisons human type II topoisomerases. Chem. Res. Toxicol..

[173] Moustakli E., Zikopoulos A., Skentou C., Katopodis P., Domali E., Potiris A., Stavros S., Zachariou A. (2024). Impact of reductive stress on human infertility: Underlying mechanisms and perspectives. Int. J. Mol. Sci..

[174] Moazamian A., Hug E., Villeneuve P., Bravard S., Geurtsen R., Hallak J., Saez F., Aitken R.J., Gharagozloo P., Drevet J.R. (2025). The dual nature of micronutrients on fertility: Too much of a good thing?. F S Sci..

[175] Aitken R.J. (2021). Antioxidant trials—The need to test for stress. Hum. Reprod. Open.

[176] Barratt C.L.R., Björndahl L., De Jonge C.J., Lamb D.J., Osorio Martini F., McLachlan R., Oates R.D., van der Poel S., St John B., Sigman M. (2017). The diagnosis of male infertility: An analysis of the evidence to support the development of global WHO guidance-challenges and future research opportunities. Hum. Reprod. Update.

[177] Pokorska-Niewiada K., Janda-Milczarek K., Kayumov K., Ziętek M., Szczuko M. (2026). Non-mineral antioxidant supplementation in endometriosis: Biological rationale, clinical evidence, and therapeutic implications-a narrative review. Nutrients.

[178] Subakathulla S., Manoj N., Patanwala A.M., Premvignesh P.A., Maher Alobaid Y., Majie A., Gorain B., Dutta S., Sengupta P., Rosas I.M. (2026). Redox-endocrine triad in PCOS: Can vitamin D, myo-inositol, and melatonin synergize as bioactive cocktails?. Front. Endocrinol..

[179] Showell M.G., Mackenzie-Proctor R., Jordan V., Hart R.J. (2020). Antioxidants for female subfertility. Cochrane Database Syst. Rev..

